# rs3764435 Associated With Parkinson's Disease in Mexican Mestizos: Case-Control Study Reveals Protective Effects Against Disease Development and Cognitive Impairment

**DOI:** 10.3389/fneur.2019.01066

**Published:** 2019-10-09

**Authors:** Alma C. Salas-Leal, Ada Sandoval-Carrillo, Elizabeth Romero-Gutiérrez, Francisco X. Castellanos-Juárez, Edna M. Méndez-Hernández, Osmel La Llave-León, Gerardo Quiñones-Canales, Oscar Arias-Carrión, José M. Salas-Pacheco

**Affiliations:** ^1^Instituto de Investigación Científica, Universidad Juárez del Estado de Durango, Durango, Mexico; ^2^Unidad de Trastornos del Movimiento y Sueño, Hospital General Dr. Manuel Gea González, Mexico City, Mexico; ^3^Hospital General Santiago Ramón y Cajal-ISSSTE, Durango, Mexico; ^4^Centro de Innovación Médica Aplicada, Hospital General Dr. Manuel Gea González, Mexico City, Mexico

**Keywords:** parkinson's disease, ALDH1A1, rs3764435, protective factor, polymorphism

## Abstract

Parkinson's disease (PD) is the second most common movement disorder. Genetic risk factors provide information about the pathophysiology of PD that could potentially be used as biomarkers. The *ALDH1A1* gene encodes for the aldehyde dehydrogenase enzyme, which is involved in the disposal of toxic metabolites of dopamine. Due to the cytotoxic nature of aldehydes, their detoxification is essential for cellular homeostasis. It has been reported that *ALDH1A1* expression levels and activity are decreased in PD patients. A deficit in ALDH1A1 activity in the substantia nigra, may lead to the accumulation of neurotoxic aldehydes and eventually the cell death seen in PD. One of the single nucleotide polymorphisms (SNP) that may modulate ALDH1A1 activity levels is rs3764435 (A/C). To investigate whether a statistical association exists between PD and the SNP rs3764435, we carried out a population-based Case-Control association study (120 PD patients and 178 non-PD subjects) in Mexican mestizos. DNA was extracted from blood samples and genotyped for rs3764435 using real-time PCR. A significant difference was found between PD cases and controls in both allelic and genotypic frequencies. The calculated OR showed that the C/C genotype is a protective factor under the codominant and recessive models of inheritance. However, after stratifying by sex, the protective role of this genotype is conserved only in men. Also, under the codominant and dominant models, rs3764435 appears to exert a protective effect against cognitive impairment in PD patients. Here for the first time, we show an association between PD and rs3764435 in a Mexican mestizo population, suggesting it confers neuroprotection for dementia in PD and is neuroprotective against developing PD in the males of this population. While analysis of the SNP looks favorable, replication of our study in cell lines or rs3764435 KO mice is required to validate these results.

## Introduction

Parkinson's disease (PD) is one of the most commonly occurring neurodegenerative and movement disorders; second only behind Alzheimer's and essential tremor, respectively (1, 2). Since neuropathological confirmation is required for a definite diagnosis of PD, its diagnosis is mainly based on clinical characteristics including, bradykinesia, rigidity, resting tremor, and postural instability. These manifestations evolve with long-standing disease and are related, in part, to loss of dopaminergic neurons in the substantia nigra pars compacta (SNpc) (3). In most cases, symptoms appear when about 60% of these neurons are lost. However, mere observation of the defining clinical criteria does not provide a specific and sensitive enough prognosis and diagnosis (4, 5). Nevertheless, structured scoring systems, such as the Unified Parkinson's Disease Rating Scale (UPDRS) (6) and Hoehn and Yahr staging scale (H&Y) (7) are useful to measure disease progression.

**Figure 1:**
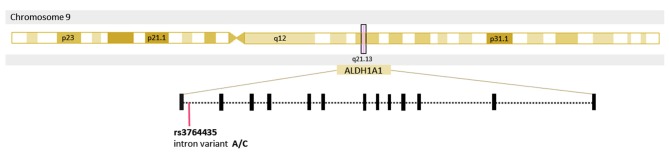
Location of the single nucleotide polymorphism rs3764435 in the gene Aldehyde dehydrogenase 1 family member A1 *(ALDH1A1)*.

To date, the etiology of PD remains unclear (8). Genetic susceptibility, environmental pollutants, chronic inflammation, among others, are considered risk factors for its development (8). As neuroprotection at the onset of PD could help prevent further progression of the disease (5), discovering an early detection method is vital. Genetic studies have shown that several mutations and polymorphisms provide relevant information about the pathophysiology of PD, and also suggest that these could potentially serve as diagnostic biomarkers. Research in different populations has led to the identification of several monogenic forms of PD and numerous genetic factors associated with the disease. Most of these genes are involved in dopamine transmission, transport, and degradation (9). *ALDH1A1* (aldehyde dehydrogenase 1 family member A1) is a protein-coding gene involved in the degradation of 3,4-Dihydroxyphenylacetaldehyde (DOPAL), a highly toxic dopamine metabolite (8–10) (Figure 1). Blaschko predicted the possible neurotoxicity of the aldehyde metabolites of amines 60 years ago, due to their highly reactive nature (11). The accumulation of DOPAL has been reported to be toxic and shown to trigger dopaminergic degeneration in mice (12). Additionally, a study with ALDH1a1 and ALDH2 knockout mice showed significant increases in biogenic aldehydes, L-DOPA-responsive and age-dependent deficits in motor performance, and death of SNpcDA neurons (12). Their findings indicate that impaired detoxification of biogenic aldehydes may cause neuronal degeneration similar to that of PD, which correlates with the significant differences in *ALDH1A1* expression levels found in peripheral blood and brain tissue of PD patient's vs. non-PD subjects (9). The single nucleotide polymorphism (SNP) rs3764435 (A to C transition) is located in the intron region of *ALDH1A1* suggesting it has an important role in modulating gene expression (13). Little is known of the genetic variants conferring risk or protection for PD in Mexican mestizos. The present study aims to determine whether an association exists between rs3764435 and PD in this population group.

## Materials and Methods

### Study Population

Mexican mestizos subjects, that is, individuals originated from the admixture of European and Native American ancestral populations, were recruited from three public hospitals in Mexico. Hospital General Dr. Manuel Gea Gonzalez in Mexico City (Central Mexico), Hospital General 450 and Hospital General Santiago Ramón y Cajal in Durango City (Northwest Mexico). All study procedures were performed in accordance with the ethics and research committees of these hospitals and with the 1964 Helsinki declaration and its later amendments. Written consent forms were signed before any intervention. A medical record was created for all subjects that included an evaluation determining or excluding the presence of PD. Two neurologists using diagnostic criteria of the United Kingdom Parkinson's Disease Society Brain Bank (UKPDSBB) confirmed PD. Hoehn & Yahr staging and the Unified Parkinson's Disease Rating Scale (UPDRS) were used for clinical evaluation (6, 7). Cognition was measured with the Mini-Mental State Examination (MMSE) (14), a cut-off for cognitive impairment below to 26 was considered. Depression was assessed using the Hamilton Depression Rating Scale (HDRS) with a cut-off below to 8 (15).

### DNA Extraction and Genotyping

Genomic DNA was extracted from whole blood using a procedure for rapid isolation of genes (16). The DNA concentration and purity were measured by a NanoDrop 2000 spectrophotometer (Thermo Fisher Scientific Inc., Germering, Germany). Genotyping of the SNP rs3764435 in *ALDH1A1* was performed with StepOne Real-Time PCR equipment (Applied Biosystems, Carlsbad, CA, USA) using a TaqMan assay (SNP ID C__27109535_10, Applied Biosystems). The polymerase chain reaction was performed to a final volume of 20 μL per well-containing 10 ng of genomic DNA, 0.625 μL of Taqman SNP genotyping assay and 5 μL of genotyping master mix. Amplification consisted of a first step at 60°C for 30 s and 95°C for 10 min followed by 40 cycles of 92°C for 15 s and 60°C for 1 min, and a final step of 60°C for 30 s.

### Statistical Analyses

We used measures of central tendency to describe data and a Student's *t*-test to compare continuous variables. A Pearson X2-test was used to compare allele and genotype frequencies. A classical χ^2^ goodness-of-fit test evaluated the Hardy-Weinberg (H-W) equilibrium. Odds ratio (OR) and 95% confidence interval (CI) were calculated with a binary logistic regression model to estimate the association between the SNP rs3764435 A/C and the risk of PD. All statistical analyses were performed using Stata software release 13 (17).

## Results

The demographic and clinical characteristics of PD patients and controls are summarized in Table 1. Statistical difference was found between the two groups' depression scores (as measured by the HDRS).

**Table 1:** Summary of subject's demographic and clinical characteristics.

	**Control**	**PD**	***p***
*Total sample*	178	120	
Males *n* (%)	90 (59.6)	61 (50.83)	0.963^§^
Age at enrollment, years	69.26 ± 8.95	70.5 ± 9.35	0.254^+^
Depression by HAM-D scores *n* (%)	78 (48.75)	81 (72.97)	**<0.001**^§^
Cognitive impairment by MMSE scores *n* (%)	47 (27.01)	36 (43.14)	0.351^§^
Age at onset of PD, years		65.29 ± 9.22	
UPDRS III		42.82 ± 20.85	
UPDRS total		72.88 ± 34.49	
Hoehn and Yahr scale *n* (%)			
≤ 2.5		44 (38.6)	
≥3		70 (61.4)	

Mean values (Standard deviation) or frequency (%), HAM-D, Hamilton Depression Rating Scale; MMSE, Mini-Mental State Exam; UPDRS, Unified Parkinson Disease Rating Scale. p values determined by

+ Student's t-test for parametric and

§ *X^2^ test for categorical data*.

^*^Bold type reflects statistically significant values.

The genotype frequencies of the control group were in H-W equilibrium (*p* = 0.789). The results of the analysis of genotype frequencies and logistic regression, adjusted by sex and age, are shown in Table 2. We found significant differences in allelic and genotypic frequencies between the PD cases and control group (*p* = 0.024 and *p* = 0.011). There was a significant association between the SNP rs3764435 and PD under the codominant and recessive models of inheritance (OR = 0.40, CI95% = 0.19–0.84 *p* = 0.016 and OR = 0.40, CI95% = 0.21–0.75 *p* = 0.005, respectively). This association remained significant after adjustment for risk factors such as smoking, alcoholism, metal and pesticide exposure, well-water consumption or recruitment site (data not shown).

**Table 2:** Allele and genotype frequencies of rs3764435 and PD risk estimation.

**Gene**	**Marker**	**Allele frequency**				**Genotypic frequency**				Odds ratio [CI 95%]^*^		
			**Control** ***n* (%)**	**PD** ***n* (%)**	***p***		**Control** ***n* (%)**	**PD** ***n* (%)**	***p***	**Models**		***p***
*ALDH1A1*	rs3764435	C^+^	187 (0.5)	103 (0.43)	**0.024**	AA	41 (0.23)	33 (0.28)	**0.011**	Codominant		
	A>C^**+**^									AA vs. AC	1 [0.58–1.76]	0.968
										AA vs. CC	0.40 [0.19–0.84]	**0.016**
	Intronic					AC	87 (0.49)	71 (0.59)		Dominant		
										AA vs. AC+CC	0.79 [0.46–1.35]	0.40
	Variant					CC	50 (0.28)	16 (0.13)		Recessive		
										AA+AC vs. CC	0.04 [0.21–0.75]	**0.005**

* Models adjusted by sex and age. Hardy-Weinberg equilibrium in controls = 0.789. ^*^Bold type reflects statistically significant values.

+ *allele of minor frequency*.

Sex-stratified analysis (Table 3) indicates, the association between this C/C genotype and a protective effect for PD is conserved under the codominant and recessive models in men (OR = 0.27, CI95% = 0.09–0.79 *p* = 0.017 and OR = 0.30, CI95% = 0.12–0.75 *p* = 0.011, respectively) but not in women (OR = 0.61, CI95% = 0.22–1.71 *p* = 0.35 and OR = 0.54, CI95% = 0.22–1.31 *p* = 0.17, respectively). This association remained even after adjusting for exposure to environmental risk factors and place of recruitment (data not shown).

**Table 3:** Allele and genotype frequencies of rs3764435 and PD risk estimation by sex.

**Allele frequency in Males**				**Genotypic frequency in Males**				Odds ratio [CI 95%]^*^		
	**Control *n* (%)**	**PD *n* (%)**	***p***		**Control *n* (%)**	**PD *n* (%)**	***p***	**Models**		***p***
C^+^	98 (54%)	50 (41%)	**0.025**	AA	19 (21%)	18 (30%)	**0.024**	Codominant		
								AA vs. AC	0.86 [0.39–1.88]	0.714
								AA vs. CC	0.27 [0.09–0.79]	**0.017**
				AC	44 (49%)	36 (59%)		Dominant		
								AA vs. AC+CC	0.64 [0.30–1.37]	0.24
				CC	27 (30%)	7 (11%)		Recessive		
								AA+AC vs. CC	0.30 [0.12–0.75]	**0.011**
**Allele frequency in Females**				**Genotypic frequency in Females**						
	**Control** ***n (%)***	**Case** ***n (%)***	***p***		**Control** ***n (%)***	**Case** ***n (%)***	***p***			
C^+^	89 (50%)	53 (45%)	0.401	AA	22 (25%)	15 (25%)	0.266	Codominant		
								AA vs. AC	1.18 [0.53–2.61]	0.68
								AA vs. CC	0.61 [0.22–1.71]	0.35
				AC	43 (48%)	35 (59%)		Dominant		
								AA vs. AC+CC	0.99 [0.46–2.12]	0.98
				CC	23 (26.2)	9 (16%)		Recessive		
								AA+AC vs. CC	0.54 [0.22–1.31]	0.17

* Models adjusted by age. ^*^Bold type reflects statistically significant values.

In addition to these results, the C variant was associated with a protective effect for cognitive impairment in the PD subjects (Table 4) under codominant and dominant models of inheritance (OR = 0.34, CI95% = 0.12–1.97 *p* = 0.045 and OR = 0.30, CI95% = 0.11–0.85 *p* = 0.024, respectively).

**Table 4:** Risk estimation for cognitive impairment of rs3764435 in PD group.

**Models**	Odds ratio [CI 95%]^*^	***p***
**Codominant**		
AA vs. AC	0.34 [0.12–0.97]	**0.045**
AA vs. CC	0.18 [0.02–1.13]	0.068
**Dominant**		
AA vs. AC+CC	0.30[0.11–0.85]	**0.024**
**Recessive**		
AA+AC vs. CC	0.35[066–1.94]	0.235

* Models adjusted by age. ^*^Bold type reflects statistically significant values.

## Discussion

The aldehyde dehydrogenase 1 gene, codified by *ALDH1A1*, has been implicated in dopamine metabolism by oxidation of DOPAL into 3,4-dihydroxyphenylacetic acid (DOPAC). DOPAL is highly reactive and is considered to be toxic; it activates the formation of oligomers and aggregates of alpha-synuclein (α-syn), and the production of reactive oxygen species (ROS) that trigger dopaminergic neuronal degeneration in the SNpc (8, 18, 19). Given the role of ALDH1A1 in the elimination of the toxic metabolites of dopamine, its expression levels were expected to be reduced in PD patients; however, results have been inconsistent in the literature. Some studies suggest a down-regulation of this gene in the SNpc and blood (9, 20, 21), others indicate its up-regulation in the blood of PD subjects compared with controls (5) while in others, no difference was observed (22). These inconsistencies could be related to several factors, including variations in the SNPs in the *ALDH1A1* gene.

In this case-control study, we evaluate the rs3764435 polymorphism of *ALDH1A1*. The SNP rs3764435 is an intronic variant reported to be a member of a haplotype that may regulate the expression of *ALDH1A1*. Our results showed that the C allele is present more frequently in non-PD subjects (*p* = 0.021) and logistic regression revealed that the C/C genotype might be acting as a protective factor that decreases the risk of PD in Mexican mestizos. To our knowledge, an association between rs3764435 and PD has not been reported in the literature. In other studies, the A/A genotype of rs3764435 was associated with increased risk of hematological toxicity after the administration of cancer chemotherapeutic drugs, whose metabolites are detoxified by ALDH1A1 (23). Furthermore, ALDH1A1 also participates in the metabolism of alcohol, and allele C of rs3764435 was found more often in alcohol-dependent individuals (13, 24). Notably, sex-stratified analysis revealed the protective effect for PD to be only conserved in men. While no published evidence suggests an SNP-sex interaction effect for rs3764435, sex differences in ALDH expression have been described (25–27). Possibly, the protective effect is only maintained in men since they have a lower expression of *ALDH1A1* than women. These reports, together with our results, allow us to suggest that the C allele of rs3764435 regulates the expression of *ALDH1A1* positively.

Interestingly, when the PD subjects' MMSE test scores for cognitive impairment were compared with their gene profile, we observed a correlation between the presence of the C/C variant and a protective effect for cognitive decline in these subjects. The most common non-motor symptoms of our PD cases were neuropsychiatric, and these directly diminish the quality of life of patients (28). Studies have demonstrated higher levels of total and oligomeric α-syn in the cerebrospinal fluid and plasma of PD patients with dementia compared with PD patients without dementia (29–31); the increase of these forms of α-syn is hypothesized to be the result of an accumulation of DOPAL.

The present study has the following limitations. Although the sample size is relatively small, a *post-hoc* power analysis showed an 85% statistical power. Even so, as mentioned in the discussion, this study is not representative of the entire Mexican mestizo population. To obtain a complete picture of the association between rs3764435 and PD in our population, further large-scale case-control studies are required that include subjects from the South and Southeast of Mexico and use a well-selected panel of ancestry informative markers for population stratification. Additionally, we do not determine the gene expression or enzymatic activity, which would have allowed us to gather more evidence regarding the physiological role of rs3764435. Furthermore, metabolic and proteomic studies are necessary to confirm the upstream effect of rs3764435 variants in this population group.

In conclusion, this case-control study in Mexican mestizos shows for the first time a neuroprotective effect for PD associated with the SNP rs3764435. Our results suggest the C allele exerts a protective effect against cognitive impairment in PD patients and neuroprotection for the development of PD in males of this genetic background.

## Data Availability Statement

The raw data supporting the conclusions of this manuscript will be made available by the authors, without undue reservation, to any qualified researcher.

## Ethics Statement

The study protocol was reviewed and accepted by the research and ethics committee of Hospital General Dr. Manuel Gea González; protocol number 49-21-2015 and was conducted per the Declaration of Helsinki. The patients/participants provided their written informed consent to participate in this study.

## Author Contributions

OA-C and JS-P designed the study. AS-L, AS-C, OA-C, and JS-P collected the data. AS-L, ER-G, OA-C, and JS-P analyzed the data and performed statistical analyses. AS-L, OA-C, and JS-P drafted the manuscript. All authors contributed to and approved the final version. JS-P, FC-J, and EM-H were involved in the experimental process. OL-L and GQ-C performed the provision of patients and laboratory samples.

### Conflict of Interest

The authors declare that the research was conducted in the absence of any commercial or financial relationships that could be construed as a potential conflict of interest.
